# Design and synthesis of a novel photoaffinity probe for labelling EGF receptor tyrosine kinases

**DOI:** 10.1080/14756366.2017.1344979

**Published:** 2017-07-18

**Authors:** You-Guang Zheng, Xiao-Qing Wu, Jun Su, Ping Jiang, Liang Xu, Jian Gao, Bin Cai, Min Ji

**Affiliations:** ^a^ College of Pharmacy, Xuzhou Medical University Xuzhou PR China; ^b^ Departments of Molecular Biosciences and Radiation Oncology, University of Kansas Lawrence KS USA; ^c^ School of Chemistry and Chemical Engineering, Southeast University Nanjing PR China

**Keywords:** Epidermal growth factor receptor, activity-based protein profiling, photoaffinity probe

## Abstract

The epidermal growth factor receptor (EGFR) and HER2 are two important tyrosine kinases that play crucial roles in signal transduction pathways that regulate numerous cellular functions including proliferation, differentiation, migration, and angiogenesis. In the past 20 years, many proteomic methods have emerged as powerful methods to evaluate proteins in biological processes and human disease states. Among them, activity-based protein profiling (ABPP) is one useful approach for the functional analysis of proteins. In this study, a novel photoaffinity probe **11** was designed and synthesised to assess the target profiling of the reactive group in the photoaffinity probe **11**. Biological evaluation was performed, and the results showed that the novel photoaffinity probe binds to EGFR and HER2 proteins and it hits targets by the reactive group.

## Graphical Abstract

**Figure:**
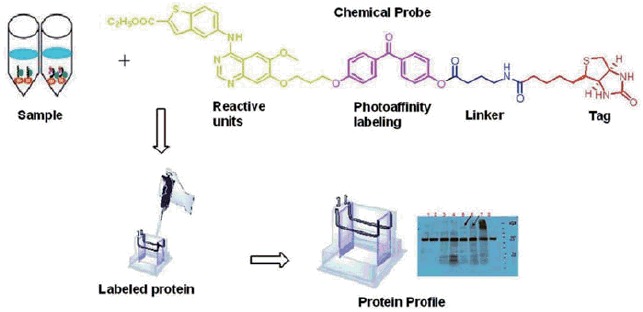

## 1. Introduction

The ERbB family of receptor tyrosine kinases consists of the epidermal growth factor receptor (EGFR) (also known as HER1/ErbB1), human EGFR2 (HER2/neu)/ERbB2, HER3/ErbB3 and HER4/ErbB4, and plays a key role in many types of solid tumors^1^. Overexpression and/or mutations of EGFR and are frequently found in many different human malignancies (e.g. breast, lung, and pancreatic cancer^2^. During the past two decades, several quinazoline derivatives targeting these two tyrosine kinases have been approved by FDA as anticancer drugs, such as Gefitinib, Erlotinib, and Lapatinib^3^. These quinazolines inhibit kinase activity of EGFR and/or HER2 mainly by occupying the ATP-binding site of the kinase.

**Figure 1.**
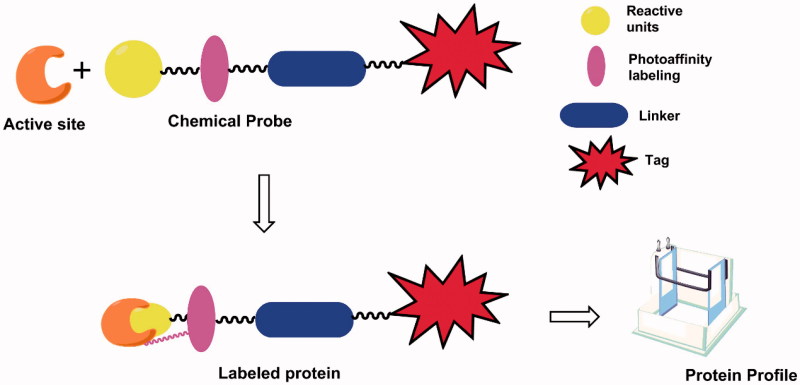
Structures and protein labelling of photoaffinity probes.

Numerous proteomic methods have emerged as powerful methods in the past 20 years to enable us to evaluate proteins in biological processes and human disease states^4–6^, including cancer^7–9^. One useful approach for the functional analysis of proteins is activity-based protein profiling (ABPP), in which probes covalently bind to the catalytic site of target proteins from cells, tissues and so on^10^ ^,^ ^11^. ABPP probes were designed to contain three key parts: (1) a reactive group for binding to enzyme active sites; (2) a reporter tag, such as a fluorophore or biotin, for detection, isolation, purification, and characterization; (3) a flexible linker^12^ ^,^ ^13^. ABPP can be classified as activity-based probes (ABPs) and affinity-based probes (AfBPs)^14^. photoaffinity labelling, as one technique of affinity-based probes, has been used to study various enzyme classes, such as kinases^15^, histone deactylases (HDACs)^16^, phosphodiesterases (PDEs)^17^, and so on (Figure 1).

**Figure 2.**
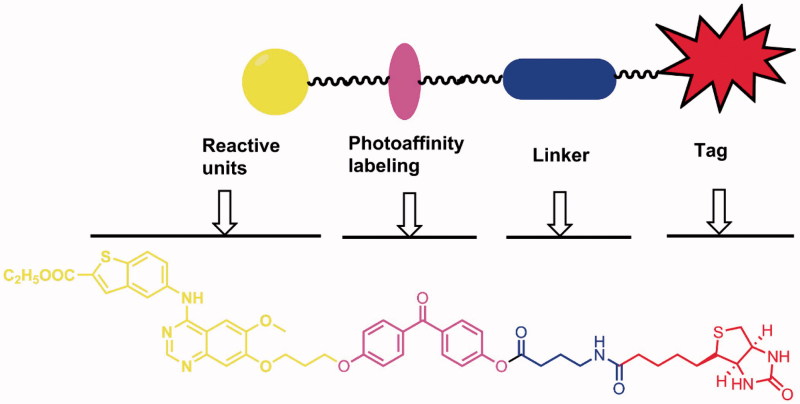
Design of the novel photoaffinity probe.

Previously, we have described the design and synthesis of two series of 4-benzothienyl amino quinazolines as new analogues of the EGFR inhibitor Gefitinib^18^. Several compounds were identified to be potent EGFR/HER2 inhibitors. To confirm the direct binding to the targets and further explore target profiling, in this article, using the shared structure of those compounds as the reactive group, we developed a novel photoaffinity probe **11** that contains a reactive group, a photoreactive group, a flexible linker, and a biotin tag (Figure 2).

## 2. Materials and methods

### 2.1. Chemical synthesis

All reagents were purchased from commercial sources and used without further purification. Melting points were measured in open capillaries and are uncorrected. ^1^H-NMR spectra were recorded in DMSO-d_6_, on Bruker Avance 300 or 500 spectrometer, or JEOL 400 spectrometer; chemical shifts (*δ*) are reported in parts per million (ppm) relative to tetramethylsilane (TMS), used as an internal standard. Mass spectra (MS) were obtained from Agilent 1100LC/MS Spectrometry Services. All compounds were routinely checked by TLC with silica gel GF-254 glass plates and viewed under UV light at 254 nm. Human pancreatic cancer cell line MIAPaCa-2 was purchased from American Type Culture Collection and cultured in high-glucose Dulbecco’s modified Eagle medium (DMEM; HyClone, Logan, UT) supplemented with 10% foetal bovine serum (FBS; HyClone) and 1% antibiotics (HyClone) in a 5% CO_2_ humidified incubator at 37 °C.

#### 2.1.1. Compounds 2–7 were prepared as described previously^18^

#### 2.1.2. Ethyl 5-((7–(3-(4–(4-hydroxybenzoyl)phenoxy)propoxy)-6-methoxyquinazolin-4-yl)amino) benzo[b]thiophene-2-carboxylate (compound 8)

Compound **7** (0.81 g, 2 mmol), bis (4-hydroxyphenyl) methanone (0.43 g, 2 mmol), K_2_CO_3_ (1.11 g, 8 mmol), catalytic amount of KI, and tetrabutyl ammonium bromide were added to 20 ml of DMF, then stirred for 36 h at 60 °C. The reaction mixture was cooled to room temperature, and then slowly poured into ice-water (200 ml). After stirred for 30 min, the precipitate was collected by filtration, which was purified by chromatographic column (silica gel 60, mesh size 200–300, dichloromethane/methyl alcohol, v/v = 75:1). The pure product was obtained as white solid (0.754 g, 58% yield). ^1^H-NMR (DMSO-d_6_, 500 MHz) *δ* (ppm): 1.35 (*t*, *J* = 4.2 Hz, 3H, –CH_2_–CH_3_), 2.29–2.34 (m, 2H, –CH_2_–CH_2_–CH_2_–), 3.98 (s, 3H, –CH_3_), 4.28 (*t*, *J* = 6 Hz, 2H, –CH_2_–CH_3_), 4.33–4.39 (m, 4H, –CH_2_–CH_2_–CH_2_–), 6.86–6.89 (m, 2H, Ar-H), 7.10–7.13 (m, 2H, Ar-H), 7.25 (s, 1H, Ar-H), 7.60–7.63 (m, 2H, Ar-H), 7.67–7.70 (m, 2H, Ar-H), 7.89 (s, 1H, Ar-H), 7.92–7.94 (dd, *J*_1_ = 13.5 Hz, *J*_2_ = 2.0 Hz, 1H, Ar-H), 8.06 (d, *J* = 9.0 Hz, 1H, Ar-H), 8.21 (s, 1H, Ar-H), 8.44 (d, *J* = 2.0 Hz, 1H, Ar-H), 8.47 (s, 1H, Ar-H), 9.66 (s, 1H, –NH–), 10.28 (s, 1H, –OH); ESI-MS *m/z*: 650.3 [M + H]^+^, 648.2 [M − H]^−^.

#### 2.1.3. 4–(5-((3aS,4 S,6aR)-2-oxohexahydro-1H-thieno[3,4-d]imidazol-4-yl)pentanamido)butanoic acid (compound 10)

Biotin **9** (0.488 g, 2 mmol) and tri-n-butylamine (0.64 ml) were added to 35 ml of DMF, then isobutyl chloroformate (0.34 ml) was added dropwise to the solution. The mixture was added to the 4-aminobutyric acid (0.206 g, 2 mmol) solution in DMF at 0 °C, and then the reaction mixture was stirred for 2 h at RT. The solvent was removed under vacuum and the product was recrystallised from alcohol afford the product as white solid (0.34 g, 52% yield). ^1^H-NMR (DMSO-d_6_, 300 MHz) *δ* (ppm): 1.39–1.59 (m, 6H), 2.02 (*t*, *J* = 7.60 Hz, 2H), 2.18 (*t*, *J* = 7.20 Hz, 2H), 2.54 (d, *J* = 12.40 Hz, 1H), 2.75–2.79 (m, 1H), 2.96–3.06 (m, 3H), 4.089 (*t*, *J* = 4.40 Hz, 1H), 4.25 (*t*, *J* = 7.60 Hz, 1H), 6.41 (d, 2H), 7.77 (s, 1H), 12.03 (s, 1H); ESI-MS *m/z*: 328.1 [M − H]^−^.

#### 2.1.4. Photoaffinity probe (compound 11)

Compound **8** (0.163 g, 0.25 mmol), compound **10** (0.08 g, 0.25 mmol), EDCI (0.25 mmol), catalytic amount of DMAP were added to 20 ml of DMF, and then stirred for 36 h at RT. The reaction mixture was poured into 200 ml ice-water, and stirred for 0.5 h. The solid formed was filtered off, purified by chromatographic column (silica gel 60, mesh size 200–300, dichloromethane/methyl alcohol, v/v = 20:1) to afford the general probe as white solid (0.086 g, 36% yield). ^1^H-NMR (DMSO-d_6_, 500 MHz) *δ* (ppm): 1.34–1.37 (m, 5H), 1.44–1.55 (m, 3H), 1.60–1.64 (m, 1H), 1.74–1.80 (m, 2H), 2.06–2.11 (dd, *J*_1 _ = 7.40 Hz, *J*_2 _ = 9.00, 2H), 2.35 (*t*, *J* = 6.20 Hz, 2H), 2.55–2.64 (m, 2H), 2.78–2.82 (dd, *J*_1 _ = 5.10 Hz, *J*_2 _ = 5.15 Hz, 1H), 3.07–3.10 (m, 1H), 3.11–3.17 (m, 2H), 4.03 (s, 3H, –CH_3_), 4.10–4.13 (m, 1H), 4.27–4.33 (m, 3H), 4.37–4.39 (m, 4H, –CH_2_–CH_2_–CH_2_–), 6.35 (s, 1H, –NH–CO–NH–), 6.40 (s, 1H, –NH–CO–NH–), –7.13 (d, *J* = 8.85 Hz, 2H, Ar-H), 7.30 (d, *J* = 8.60, 2H, Ar-H), 7.38 (s, 1H, Ar-H), 7.74 (d, *J* = 8.70 Hz, 4H, Ar-H), 7.84–7.86 (m, 2H, Ar-H, –NH–CO), 8.16 (d, *J* = 8.80 Hz, 1H, Ar-H), 8.24 (d, *J* = 7.85 Hz, 2H, Ar-H), 8.31 (d, *J* = 1.80, 1H, Ar-H), 8.77 (s, Ar-H, 1H,), 11.25 (s, 1H, –NH–); ESI-MS *m/z*: 961.3 [M + H]^+^, 995.5 [M + Cl]^−^.

### 2.2. Biological evaluation

The pull down assay was carried out using the Immunoprecipitation Kit (Roche Diagnostics, Basel, Switzerland) following the manufacturer’s instructions with minor modifications. Briefly, MIAPaCa-2 cells were lysed using lysis buffer. After precleared with streptavidin agarose (Invitrogen, Carlsbad, CA), the lysis was split equally into several parts and incubated with DMSO control or the probe with or without competitor for 30 min, followed by UV exposure at 365 nm for 60 min or not. Streptavidin agarose was then used to pull down bound proteins by incubation the agarose with the treated cell lysate overnight. After washing the streptavidin agarose with washing buffer for several times, 30 μl of gel-loading buffer was added to each agarose pellet and then samples were heated at 95 °C for 5 min. After removal of agarose by centrifuge, the samples were ready for western blot. Western Blot was performed as we reported previously^18^ using streptavidin-HRP (Thermo Scientific, #21126, Waltham, MA), or EGFR (Cell Signaling Technology, #2232, Boston, MA), and HER2 (Cell Signaling Technology, #4290, Boston, MA) antibodies.

### 2.3. Molecular dockings

The FlexX-Dock module of Sybyl version 7.1 software (Tripos Associates Inc., St. Louis, MO) was used for molecular docking study, which is used for predicting the ligand-receptor interaction modes and for hit identification by structure-based virtual screening. This programme allows ligand structures to dock in a conformationally flexible manner to a protein and adopts a rigid-body protein approximation to speed up the calculation of binding free energy.

## 3. Results

### 3.1. Synthesis of probes

**Scheme 1.**
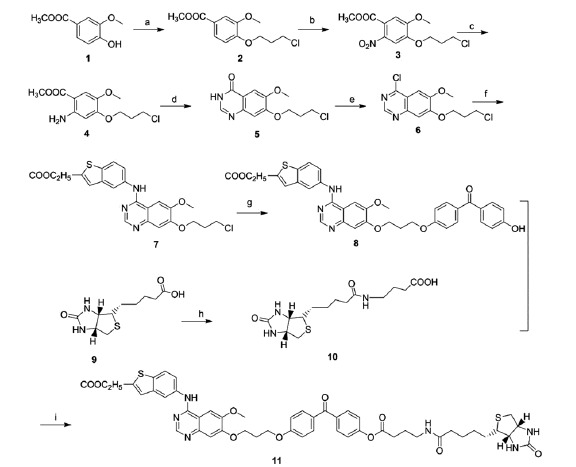
The preparation of the photoaffinity probe. Reagents and conditions: (a) 1-bromo-3-chloropropane, Potassium carbonate, DMF, 70 °C; (b) Nitric acid, acetic acid, acetic anhydride, 0–5 °C; (c) Pd/C, methanol, rt; (d) Formamidine acetate, alcohol, reflux; (e) thionyl chloride, DMF, reflux; (f) ethyl 5-aminobenzo[b]thiophene-2-carboxylate, isopropanol, reflux; (g) 4,4-dihydroxybenzophenone (DHBP), Potassium carbonate, DMF, 60 °C; (h) γ-aminobutyric acid, isobutyl chlorocarbonate, DMF, rt; (i) EDCI/DMAP, DMF, rt.

The preparation of the photoaffinity probe is illustrated in Scheme 1. Compound **7** was obtained as we described previously^18^. Compound **8** was prepared by the reaction of compound **7** with bis (4-hydroxyphenyl) methanone. Biotin **9** reacted with 4-aminobutanoic acid in N, N-Dimethylformamide (DMF) to give compounds **10**, which then coupled with compound **8** to give the general probe **11**.

### 3.2. Biological evaluation of photoaffinity probe

**Figure 3.**
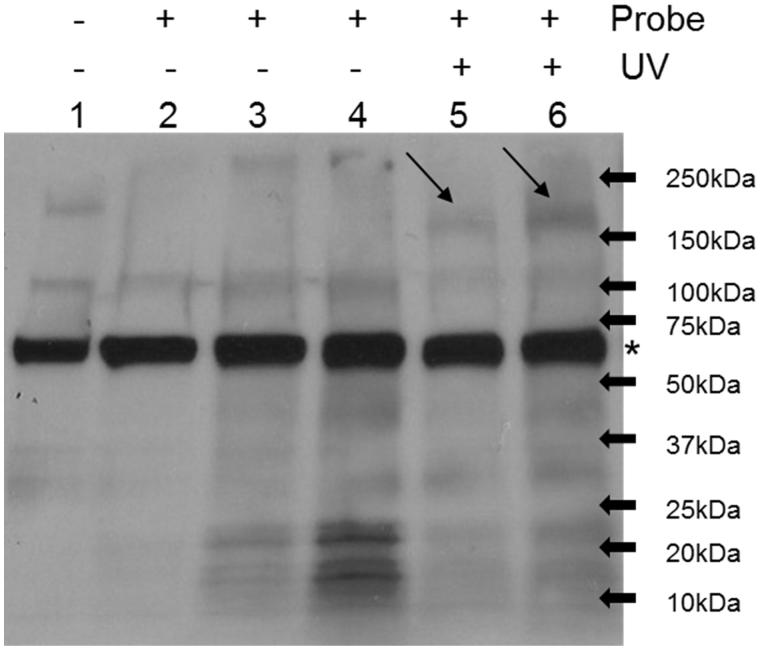
Photoaffinity labelling of cell lysate by synthesised photoaffinity probe. Lane 1: whole cell lysate; Lane 2: DMSO treatment; Lane 3: 5 μM probe treatment; Lane 4: 10 μM probe treatment; Lane 5: 5 μM probe treatment plus UV exposure; Lane 6: 10 μM probe treatment plus UV exposure. MIAPaCa2 cell lysate was treated as indicated and pull-down assay was then performed using streptavidin agarose. The samples were then run western blot and probed with streptavidin-HRP antibody. Arrows point out specific bands of streptavidin blot. * Non-specific bands.

To assess the target-binding affinity of the synthesised photoaffinity probe **11**, we first carried out pull-down assay using MIAPaCa-2 cell lysate treated with probe or DMSO control under UV exposure or not. The samples were then run Western Blot and probed with Horseradish peroxidase (HRP) conjugated Streptavidin. As shown in Figure 3, only under UV exposure condition, 5 or 10 μM probe treated samples have specific bands around 200 kDa. And the intensity of the bands is dose-dependent of the probe. As reported previously^18^, the reactive group of the probe is an EGFR/HER2 inhibitor and the bands are at right size of EGFR/HER2 proteins, we predicted the bands are EGFR and/or HER2.

**Figure 4.**
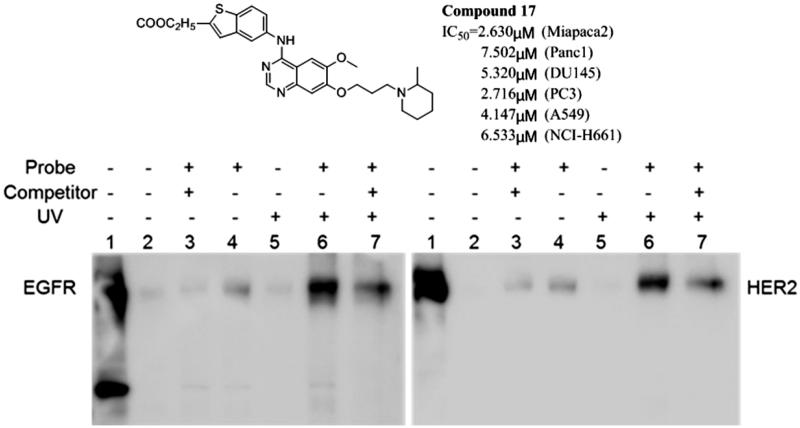
Synthesised photoaffinity probe binds to EGFR and HER2 proteins. Lane 1: whole cell lysate; Lane 2: DMSO treatment; Lane 3: 10 μM probe and 100 μM compound 17 co-treatment; Lane 4: 10 μM probe treatment; Lane 5: DMSO treatment plus UV exposure; Lane 6: 10 μM probe treatment plus UV exposure; Lane 7: 10 μM probe and 100 μM compound 17 co-treatment under UV exposure. MIAPaCa2 cell lysate was treated as indicated and pull-down assay was then performed using streptavidin agarose. The samples were then run western blot and probed with anti-EGFR (left) or anti-HER2 (right) antibody.

To confirm our prediction, a similar pull-down assay was performed and the samples were run Western Blot and probed with EGFR and HER2 antibodies, respectively. As shown in Figure 4, probe but not DMSO treated samples show bands of EGFR and HER2. And the intensity of the bands increases under UV exposure condition. More importantly, the binding of the probe is partially blocked by 10-fold more label-free the compound **17**, which contains same reactive group as the probe and is used as a competitor^18^. The compound **17** inhibited 70% of EGFR enzyme activity at 10 μM. These results indicate that the novel photoaffinity probe has high target-binding potency and it hits targets by the reactive group.

### 3.3. Molecule docking

**Figure 5.**
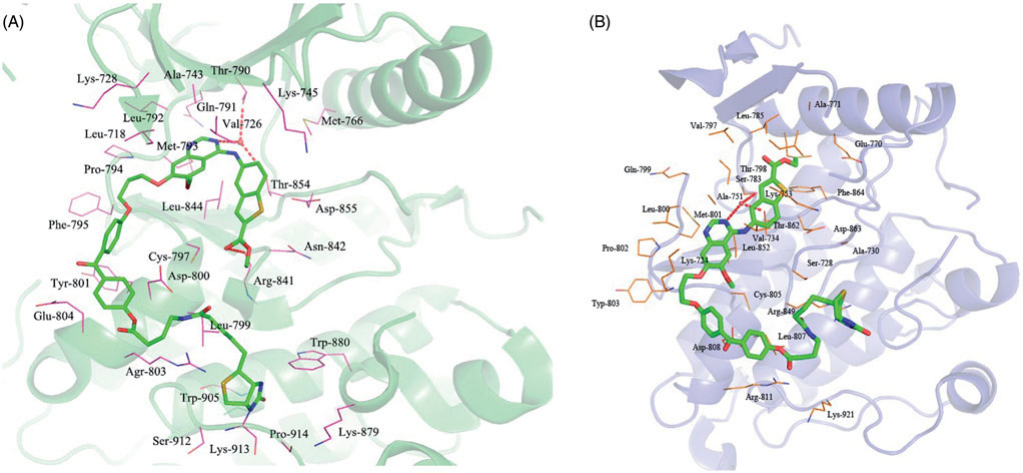
Binding models of photoaffinity probe target into active site of EGFR (A) and HER2 (B).

To understand the interaction between photoaffinity probe and kinases, the possible binding modes of photoaffinity probe on EGFR (PDB code: 2ITY) and HER2 (PDB code: 3PP0) were explored using the Sybyl 7.0. As shown in Figure 5(A), the N3 of quinazoline ring hydrogen bonds with the main chain amide of Thr790 and Thr 854 through a well-defined water molecule. Besides, the ester group in the 2 position of the benzo[b]thiophene ring is engaged in hydrogen bonding to Arg841. The binding model of photoaffinity probe into the binding site of HER2 is depicted in Figure 5(B). In this binding model, the N3 of quinazoline ring forms two hydrogen bonds with Ser783 and Thr862 via a bridging water molecule. Meanwhile, there are several hydrophobic interactions as well formed between the probe and the kinase molecules.

## 4. Discussion

To synthesis of compound **8**, three series agents as K_2_CO_3_/DMF, CH_3_CH_2_ONa/CH_3_CH_2_OH, and NaH/DMF were used to investigate O-alkylation reaction. The result shown that there was no new product obtained in CH_3_CH_2_ONa/CH_3_CH_2_OH system as well as in NaH/DMF at rt. At 60 °C, compound **8** was got with very slow rate of reaction. Basis on the above, we have added the catalytic amount of the phase transfer catalyst in this reaction, and the reaction is complete after 36 h.

Two condensation agents as DCC/DMAP and EDCI/HOBt were investigated to synthetise the photoaffinity probe **11**. The result showed that there was no photoaffinity probe **11** in the DCC/DMAP method, and the yield of the photoaffinity probe is also very low in the EDCI/HOBt condensation reaction. Finally, we found out a preferable condensation agent as EDCI/DMAP over repeated trials. The reaction is completed and the yield is relatively high when we use EDCI/DMAP as condensing agent.

In this study, we used the synthesised photoaffinity probe to confirm that the reactive group hits EGFR and HER2. Besides target validation, photoaffinity probe can also be used to explore target profiling by combing with qualitative proteomic analysis, which is the future direction of this project.

Molecular docking established the interaction of photoaffinity probe **11** with EGFR and HER2. The interactions of photoaffinity probe **11** in the active site of the EGFR are shown in Figure 5(A). The quinazoline ring is oriented in the back of the ATP-binding pocket, where it hydrogen bonds with Thr790 and Thr 854 through a well-defined water molecule. Meanwhile, there is an additional hydrogen bond between ester group of the benzo[b]thiophene ring and Arg841, which may increase affinity of photoaffinity probe **11**. The reactive group of photoaffinity probe **11** occupies the ATP active site of the kinase, and the quinazoline ring engaged in hydrogen bonding to Ser783 and Thr862. The nice binding model of photoaffinity probe **11** with EGFR and HER2 indicates that photoaffinity probe **11** has high target-binding potency.

## 5. Conclusions

In summary, we have designed and synthesised a novel photoaffinity probe **11**. Biological experiments were carried out to assess the target-binding affinity, and the results indicated that the novel photoaffinity probe has high target-binding potency and it hits targets by the reactive group. The docking studies showed that the photoaffinity probe **11** can binds to EGFR and HER2 through hydrogen bonds as well as several hydrophobic interactions.

The spectrum data for compounds **8**, **10** and **11** are available in the Supplementary material.

## Supplementary Material

IENZ_1344979_Supplementary_Material.pdf
