# Supplementary material for: Design and synthesis of a novel photoaffinity probe for labelling EGF receptor tyrosine kinases
Source: J Enzyme Inhib Med Chem. 2017 Jul 18;32(1):954–9. doi: 10.1080/14756366.2017.1344979 (PMC6009917; doi:10.1080/14756366.2017.1344979)
Supplement: IENZ_1344979_Supplementary_Material.pdf [file IENZ_A_1344979_SM0875.pdf]

Compound 8

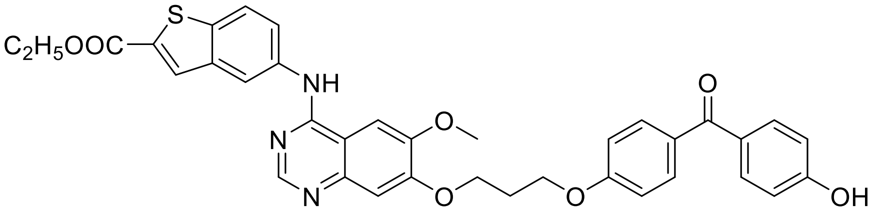

C2H5OOC
S
NH
N
N
O
O
O
O
OH

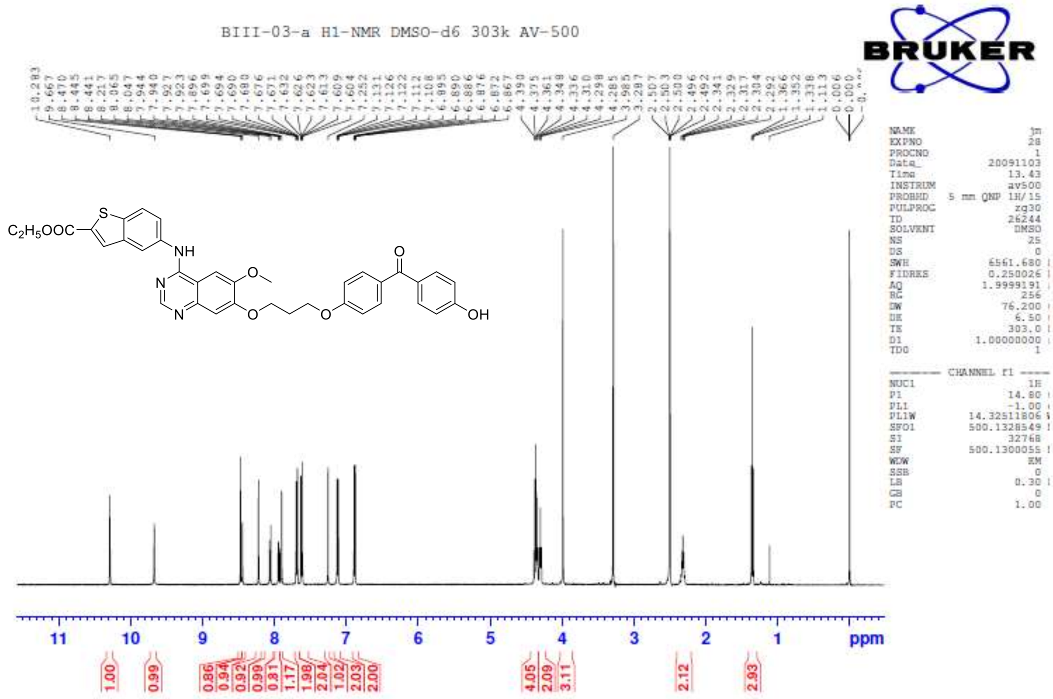

BIII-03-a H1-NMR DMSO-d6 303k AV-500
11
10
9
8
7
6
5
4
3
2
1
ppm
1.00
0.99
0.86
0.94
0.92
0.99
0.99
0.81
1.17
1.98
2.04
1.02
2.03
2.00
4.05
2.09
3.11
2.12
2.93

Display Report - Selected Window Selected Analysis

Analysis Name: BIII--02--a.d
Method: Copy of Xu.MS
Sample Name: Dummy
Analysis Info:

Instrument: LC-MSD-Trap-SL
Operator: Administrator

Print Date: 11/03/09 15:25:23
Acq. Date: 11/03/09 15:18:24

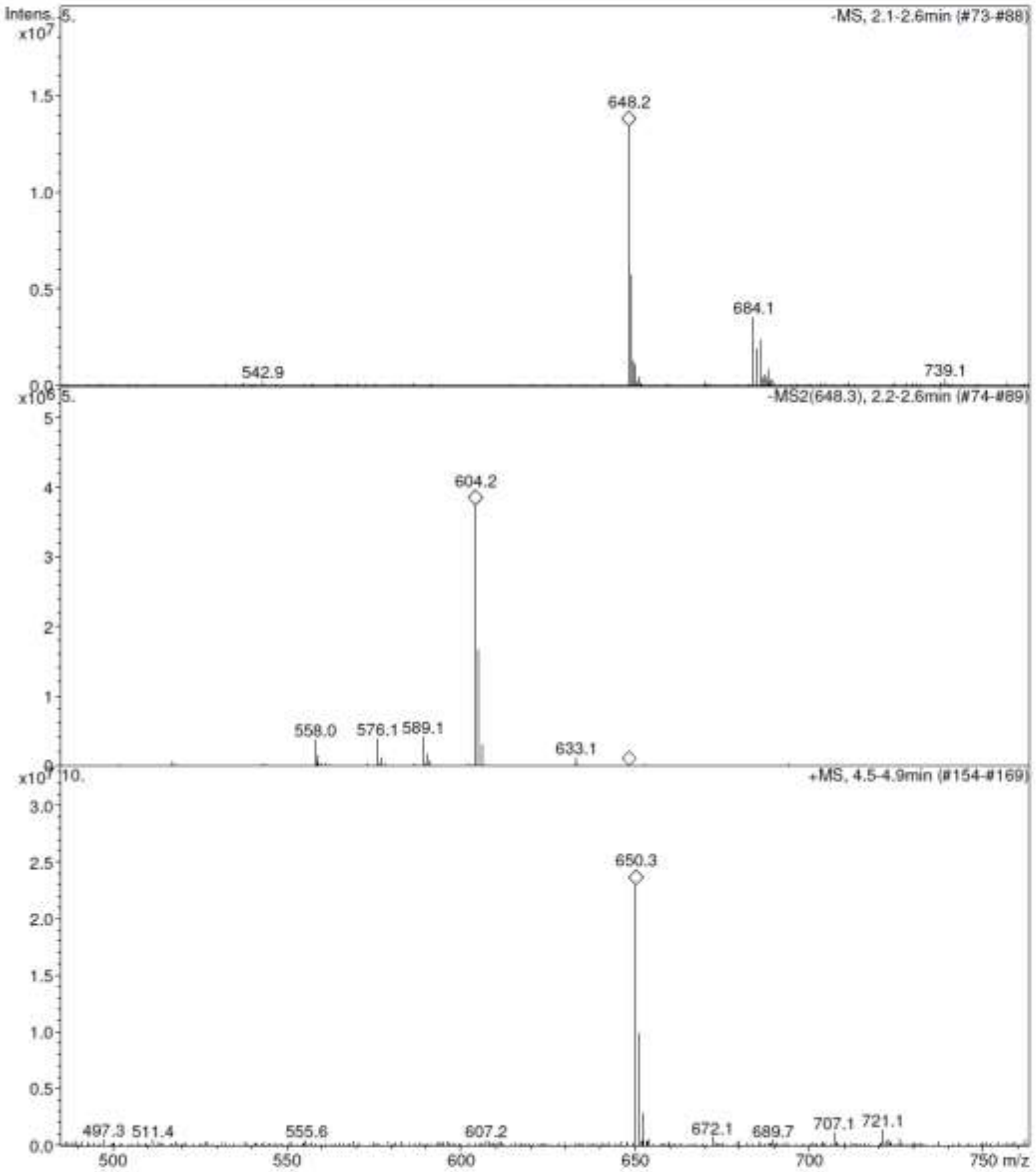

Compound 10

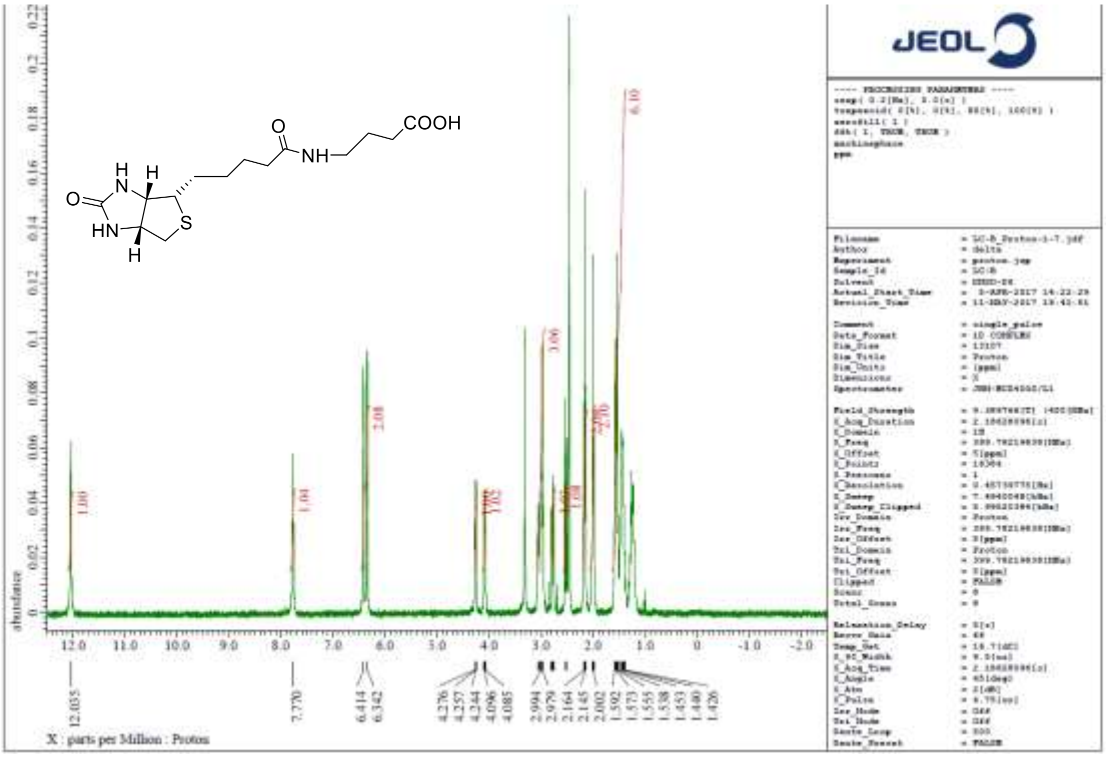
JEOL
COOH
NH
O
H
N
HN
S
abundance
X : parts per Million : Proton
12.035
7.770
6.414
6.342
4.276
4.257
4.244
4.096
4.085
2.994
2.979
2.164
2.145
2.002
1.592
1.573
1.555
1.538
1.453
1.440
1.426
1.00
1.04
2.08
3.06
6.10

Compound 11

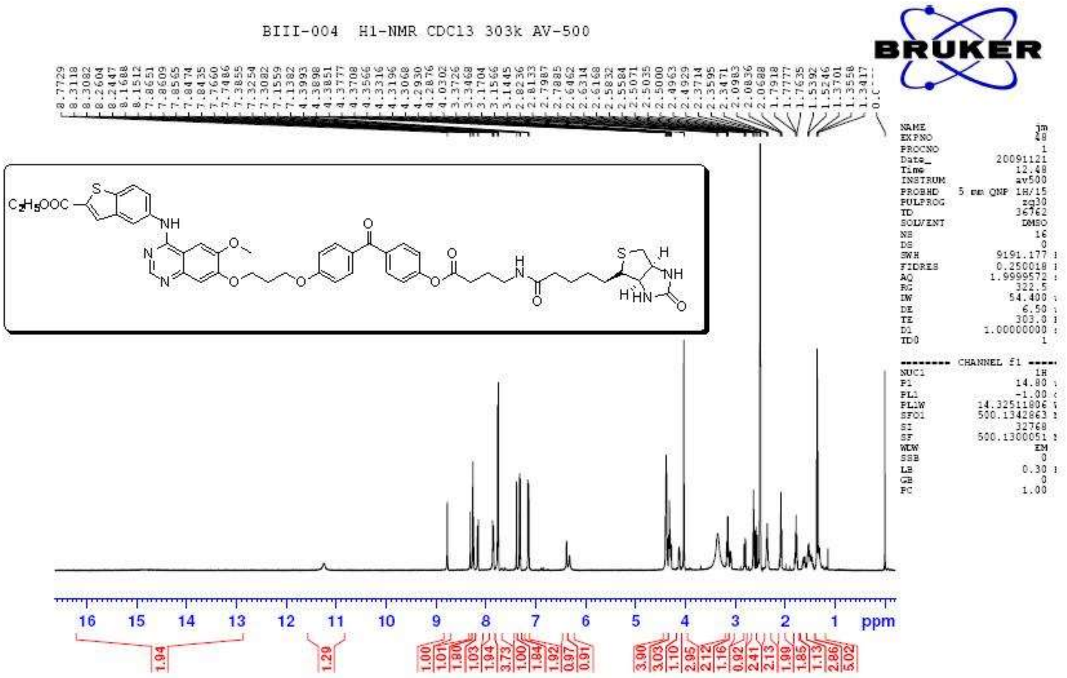
BIII-004 H1-NMR CDCl3 303k AV-500
BRUKER
ppm

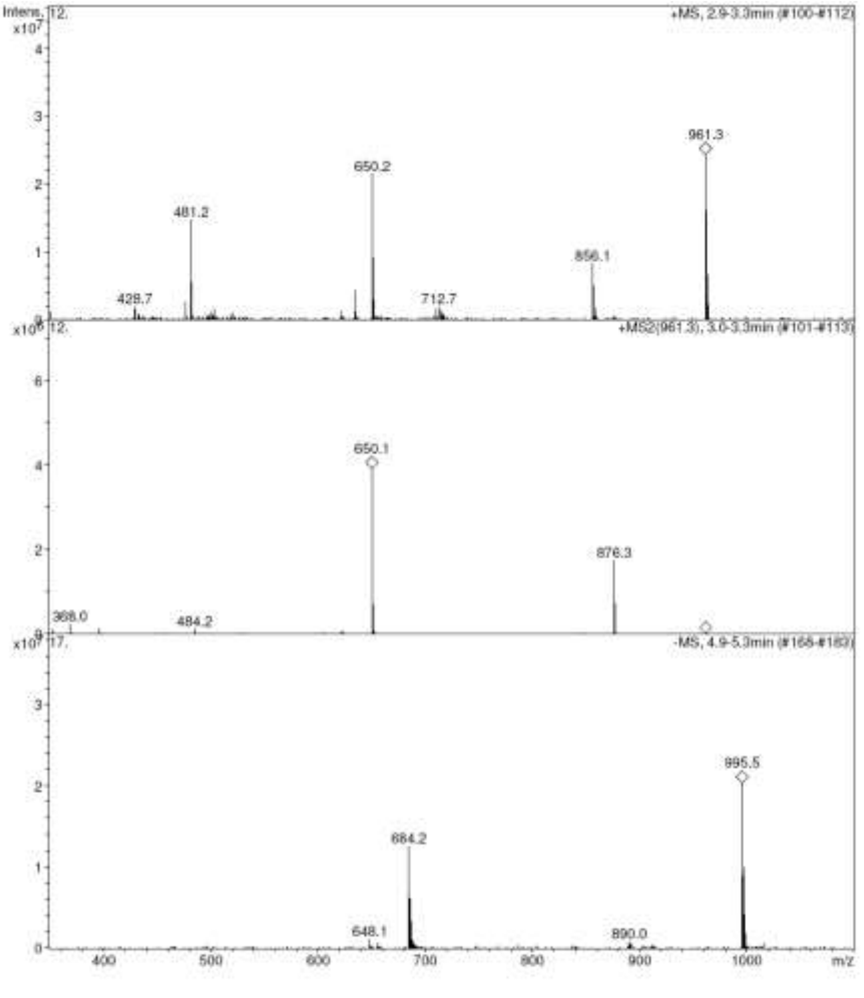
+MS, 2.9-3.3min (#100-#112)
961.3
650.2
481.2
856.1
428.7
712.7
+MS2(961.3), 3.0-3.3min (#101-#113)
650.1
876.3
368.0
484.2
-MS, 4.9-5.3min (#168-#183)
995.5
684.2
648.1
890.0
m/z
